# Dendrobine promotes bone formation via the canonical Wnt/β-catenin signaling pathway and prevents postmenopausal osteoporosis

**DOI:** 10.3389/fphar.2025.1616070

**Published:** 2025-12-03

**Authors:** Shengfa Li, Peili Liang, Jian Chen, Youming Zhang, Haixiong Miao, Ting Wang

**Affiliations:** 1 The Third People’s Hospital of Chengdu, Chengdu, Sichuan, China; 2 Department of Obstetrics and Gynecology, Center for Reproductive Medicine/Department of Fetal Medicine and Prenatal Diagnosis/BioResource Research Center, Guangdong Provincial Key Laboratory of Major Obstetric Diseases, The Third Affiliated Hospital of Guangzhou Medical University, Guangzhou, Guangdong, China; 3 Department of Orthopedics, Three Gorges Affiliated Hospital of Chongqing Medical University, Chongqing, China; 4 Chongqing Three Gorges Central Hospital, Chongqing, China; 5 Department of Chemical Engineering, Tsinghua University, Beijing, China; 6 Department of Orthopedics, Guangzhou Red Cross Hospital, Jinan University, Guangzhou, Guangdong, China

**Keywords:** osteoporosis, dendrobine, molecular biology, Wnt/β-catenin, GSK-3β

## Abstract

**Background:**

In early postmenopausal women with sex steroid deficiency, an imbalance between bone resorption and bone formation causes an accelerated decline in bone mineral density due to a longer lifespan of osteoclasts and a shorter lifespan of osteoblasts. Given this context, dendrobine, as a primary bioactive alkaloid isolated from *Dendrobium nobile* Lindl., has potential efficacy on osteoporosis due to the traditional use of *Dendrobium nobile* Lindl. in musculoskeletal disease.

**Objective:**

To investigate the anti-osteoporotic efficacy of dendrobine in increasing osteoblastogenesis and to elucidate the underlying mechanism.

**Methods:**

Using C3H/10T1/2 cells and human bone marrow-derived mesenchymal stromal cells, the effects of dendrobine on osteoblastogenesis and underlying mechanism were assayed by Western blotting, alkaline phosphatase staining, and quantitative polymerase chain reaction. The effectiveness of dendrobine in preventing postmenopausal osteoporosis was assessed using an ovariectomized mouse model by micro-computed tomography, H&E, alkaline phosphatase, and immunohistochemical staining.

**Results:**

In addition to inducing alkaline phosphatase, Runx2, osteocalcin, and Osterix expression in C3H/10T1/2 cells or human bone marrow-derived mesenchymal stromal cells, Dendrobine increased Runx2, alkaline phosphatase, and osteocalcin expression in ovariectomy (OVX) mice.

**Conclusion:**

In OVX mice, dendrobine attenuated the loss of cancellous bone, as shown in micro-computed tomography analysis. Dendrobine activated the Wnt/β-catenin pathway by suppressing the phosphorylation level of GSK-3β at Tyr216 site while keeping the level of β-catenin sustaining. This study suggests that dendrobine may be an effective treatment for postmenopausal osteoporosis.

## Graphical Abstract

**Figure:**
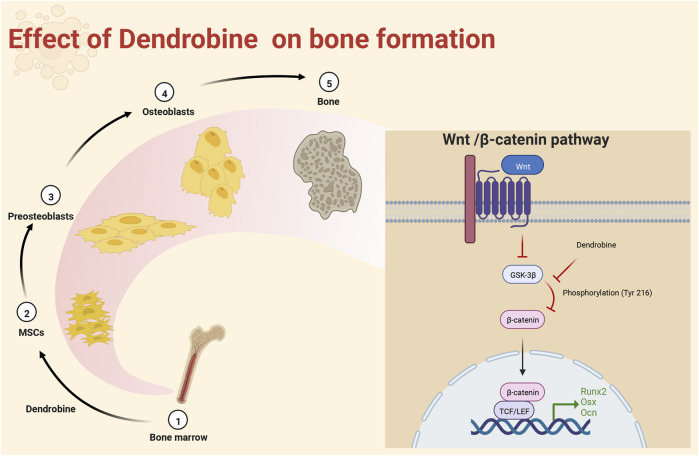
Dendrobine promotes bone formation by inhibiting the phosphorylation of GSK-3β at Tyr216 via the canonical Wnt/β-catenin signaling pathway.

## Highlights

- Dendrobine induced MSCs osteogenic differentiation
- Dendrobine attenuated postmenopausal osteoporosis by promoting bone formation
- Dendrobine suppressed GSK-3β phosphorylation to increase Wnt/β-catenin signaling

## Introduction

Osteoporosis is a chronic condition in which bone minerals become depleted, and the bones become microstructurally deteriorated (Compston et al., 2019). As a result of declining ovarian function and the downregulation of estrogen (E2) levels, postmenopausal women are susceptible to osteoporosis (Cheung et al., 2016). Which further enhances the function of osteoclasts and increases the formation of cancellous bone insufficient to compensate for increasing trabecular bone resorption, leading to postmenopausal osteoporosis (Almeida et al., 2017). Additionally, E2 not only inhibits the resorption of cancellous bone but also stimulates the formation of cancellous bone (Chow et al., 1992). The E2 deficiency prolongs the life expectancy of osteoclasts but decreases the osteoblasts’ life expectancy (Manolagas, 2010).

The treatment strategy for osteoporosis involves inhibiting osteoclasts to reduce bone resorption and/or increasing osteoblasts to increase bone formation. Currently most current osteoporosis therapies target osteoclasts, so there are only a few agents that specifically target osteoblasts (An et al., 2016). Anti-osteoporosis drugs like teriparatide stimulate osteoblast formation and inhibit osteoblast apoptosis. However, parathyroid peptides have been shown to increase osteosarcoma incidence in rats (Watanabe et al., 2012). Prolonged use of these drugs for an extended period can also damage the cortical bone structure (McClung, 2017). In recent years, the gap in clinical osteoporosis treatment has widened due to serious side effects, poor efficacy, and high prices of anti-osteoporosis drugs (Lovato and Lewiecki, 2017). Therefore, it is critical to find another anti-osteoporosis medicine that is safer, cheaper, and more effective (Khosla and Hofbauer, 2017).

As a primary bioactive alkaloid, dendrobine (DE) was first isolated in 1932 (Ci et al., 2020) from *Dendrobium nobile* Lindl., the raw plant material of this traditional Chinese herb (Deng et al., 2022). As documented in the Chinese Pharmacopoeia (National Pharmacopoeia Committee, 2010), DE has been used for discrimination and quality control of dendrobium (Wang et al., 2016). Several studies have demonstrated that DE protects against Parkinson’s disease by inhibiting apoptosis in dopaminergic neurons (Li et al., 2022), alleviates gestational diabetes mellitus (GDM) in mice (Feng et al., 2021), has antiviral properties against influenza A viruses (Li et al., 2017), induces cancer cell-specific cell death (Song et al., 2019), and ameliorates mouse liver injuries induced by INH and RIF15 (Ci et al., 2020). DE inhibits osteoclastogenesis by reducing ROS, p38-c-Fos, and NFATc1-MMP9 *in vitro*, thus reducing inflammatory osteolysis in the LPS-induced inflammatory osteolysis mouse model (Deng et al., 2022). However, the osteoblastogenesis role of DE in postmenopausal osteoporosis remains unknown.

The inhibition of GSK-3β leads to the upregulation of Wnt/β-catenin signaling, which in turn promotes bone formation. This occurs because GSK-3β inhibition activates the Wnt signaling pathway, resulting in β-catenin accumulation in the cytoplasm, which translocates into the nucleus later to form a complex with the T cell factor/lymphoid enhancing factor (TCF/LEF) transcriptional factor family to regulate the expression levels of specific downstream genes, thus promoting the expression of osteoblast-specific genes, thereby mediating osteoblastic differentiation (Wong, 2024; AlMuraikhi et al., 2023). DE can preserve cognitive function, alleviate neuronal and synaptic defects, and improve APP/tau pathology in 3 × Tg-AD mice. DE treatment for 7 months repressed GSK3β activation, thus ameliorating tau hyperphosphorylation (Zhang et al., 2024). However, it remains unclear whether DE promotes bone formation by inhibiting GSK3β.

To investigate whether DE exerts its anti-osteoporotic effects through the canonical Wnt/β-catenin pathway, we designed a combined cellular and animal study. We hypothesized that DE mitigates cancellous bone loss in OVX mice by enhancing osteoblastogenesis via activation of the canonical Wnt/β-catenin pathway. To test this, we utilized the reliable MSC model C3H/10T1/2 cells (Li et al., 2016), human bone marrow-derived mesenchymal stromal cells (hBMSCs) alongside a well-established OVX mouse model of postmenopausal osteoporosis (Chen et al., 2023).

## Materials and methods

### Materials and reagents

**FIGURE 1:**
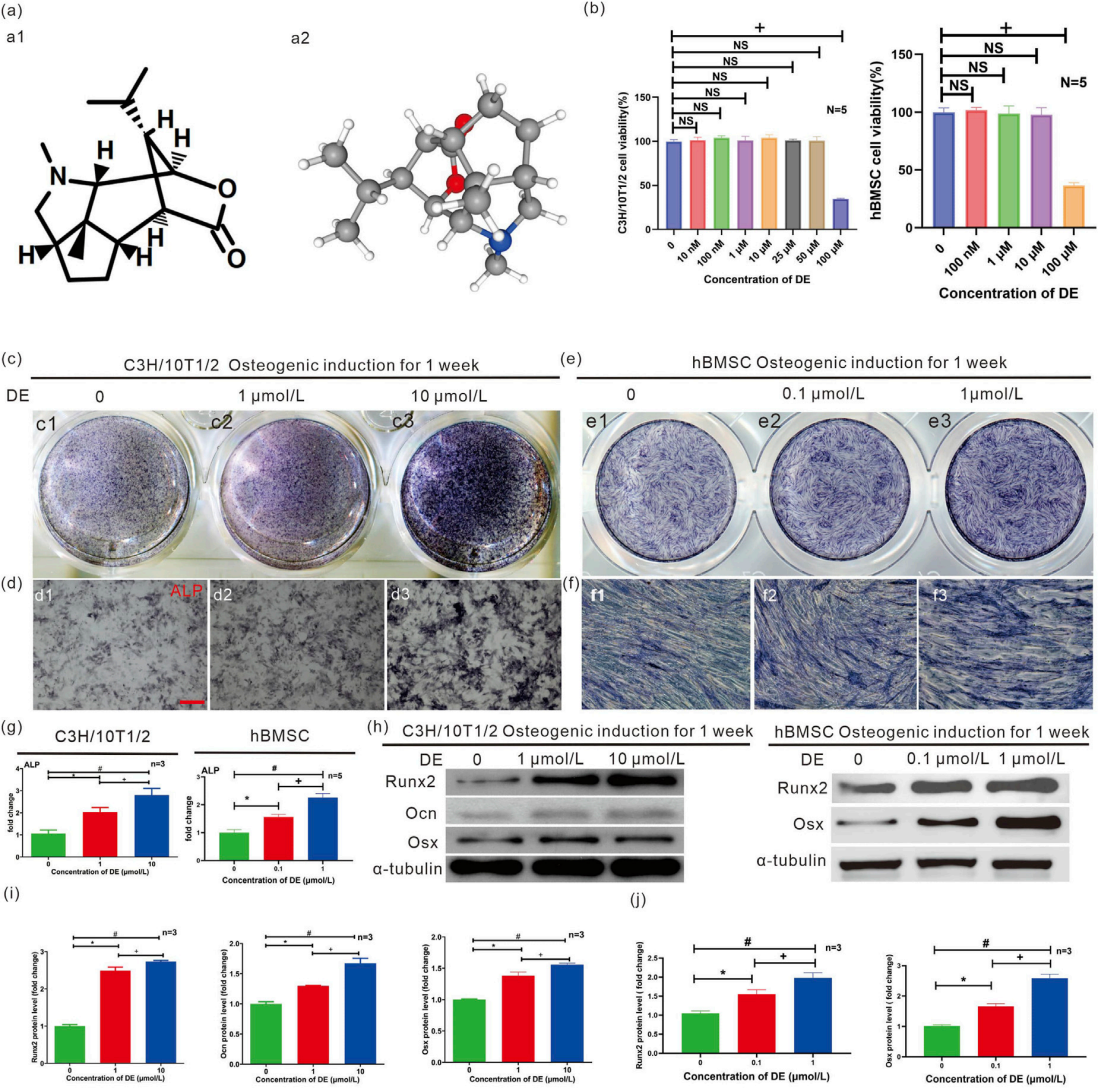
Effects of Dendrobine (DE) on C3H/10T1/2 cells and hBMSC osteogenesis differentiation *in vitro*. The osteogenic protein levels of C3H/10T1/2 cells and hBMSC were measured in the presence of DE (1 μM and 10 μM) (0.1 μM and 1 μM) for 7 days of osteogenesis-induced culture. **(a)** Molecular structure of DE in 2-dimensional (a1) and 3- dimensional style (a2). **(b)** CCK-8 assays were performed in an ordinary complete medium for 7 days to measure the effect of DE on C3H/10T1/2 cells and hBMSC’ proliferation. The clusters **(c)** and micrograph **(d)** of ALP staining assay of C3H/10T1/2 cells were performed in osteogenic-induced culture for 7 days. The clusters **(e)** and micrograph **(f)** of ALP staining assay of hBMSC were performed in osteogenic-induced culture for 7 days **(g)** Quantitative counting of the positive cell of ALP staining was performed during osteogenic-induced culture for 7 days **(h)** Western blotting (WB) was performed to measure the protein expression of Runx2, Ocn, and Osx in osteogenesis-induced cultured C3H/10T1/2 cells or hBMSC for 7 days. The quantitative gray value of WB bands from C3H/10T1/2 **(i)** and hBMSC **(j)** was measured. Quantitative counting of the positive cell was measured by Image‐Pro Plus (IPP) analysis software. The quantitative gray value of WB bands was measured by ImageJ analysis software. Each sample was measured in triplicate, and the experiment was repeated twice with similar results. CCK8:+P < 0.05 versus 100 μM DE group. C3H/10T1/2 cells: *P < 0.05 versus group without DE; +P < 0.05 versus 1 μM DE group; #P < 0.05 versus 10 μM DE group; hBMSC: *P < 0.05 versus group without DE; +P < 0.05 versus 0.1 μM DE group; #P < 0.05 versus 1 μM DE group; NS:non significance. Values were shown as mean ± SD data from independent experiments **(b,g,i,j)**.

DE (Figure 1a, molecular weight = 263.38 Da, Product No.: HB-01665, a purity of > 98% (w/w)) was purchased from Huashite Biology (Huashite Industrial Biotechnology Co., Ltd., Wuhan, Hubei, China). It was prepared in dimethyl sulfoxide (DMSO, Product No.: D2650-50ML, Sigma-Aldrich, St Louis, MO, USA) and stored at −20 °C. Phosphate-buffered saline (PBS, Product No.: P4417-100TAB), paraformaldehyde (Product No.: 158,127-3 KG), 17b-Estradiol (E2; product number: E8875), ethylenediaminetetraacetic acid (EDTA, Product No.: EDS-1KG), NaOH (Product No.: S8045-1 KG), glycerol (Product No.: G5516-4L), hematoxylin (Product No.: HHS16), and eosin (Product No.: HT110116), were purchased from Sigma-Aldrich. The cell counting kit-8 (CCK-8; Product No.: C0038) and alkaline phosphatase (ALP) staining kit (Product No.: P0321S) were obtained from Beyotime Biotechnology (Beyotime Institute of Biotechnology Co, Ltd, Shanghai, China). Minimal essential medium (MEM, Product No.: 11,095-072) and fetal bovine serum (FBS, Product No.: 10,099-141) were purchased from Gibco (Thermo Fisher Scientific Inc., Grand Island, NY, USA). L-lysine (Product No.: B877236) and sodium periodate (Product No.: S817518) were obtained from Macklin reagent (Macklin Biochemical Co., Ltd, Shanghai, China). The PrimeScript™ RT reagent kit (Product No.: RR047A) and RNAiso Plus (Product No.: 9109) and were obtained from TaKaRa (Takara Biomedical Technology Co., Ltd., Otsu, Shiga, Japan). Other chemical reagents were purchased from Sigma–Aldrich.

## Animals and DE treatment

Eight-week-old female C57/BL6 mice (body weight, 20–23 g) were obtained from the Laboratory Animal Center of Southern Medical University (44002100013267, Guangzhou, Guangdong, China). A random grouping of mice was conducted into sham, OVX, OVX + DE, and OVX + E2 groups, with ten mice per group. For the sham group, the ovaries were only surgically removed with some fat tissue but not resected. After ovariectomy, the OVX + DE group was orally administered 10 and 20 mg/kg/day of DE or intraperitoneally injection of E2 (5 mg/kg/day, used as a positive control) for 30 days. As a negative control group, saline solution was given intragastrically to sham and OVX mice. Under constant temperatures and humidity conditions, the mice were given a normal diet and water. A 12-h light and 12-h dark cycle were used for all the animals. The laboratory animal welfare program of Southern Medical University was followed during the care of the mice. Institutional animal ethics committee of Southern Medical University (Ethic No.: 20000291241) approved all *in vivo* experiments.

### Micro-computed tomography (micro-CT) analyses

The mice were anesthetized with sodium pentobarbital (40 mg/kg) and sacrificed by cervical dislocation. Bone samples were fixed in 4% (w/v) paraformaldehyde overnight. The distal femora were measured by micro-CT scan (Scanco Medical, Brüttisellen, Zurich, Switzerland) with a voltage of 55 kVp, an intensity of 200 mA, a resolution of 5 mm per pixel and an exposure of 230 m. The three-dimensional structure and morphometry were reconstructed by three-dimensional model visualization software (mCT Ray V4.2) and data analyzed by data analysis software (mCT Evaluation Program V6.6) for trabecular bone volume fraction (BV/TV), trabecular thickness (Tb. Th), trabecular number (Tb. N), trabecular separation (Tb. Sp).

### Immunohistochemical (IHC) staining

Bone specimens were fixed with 4% paraformaldehyde overnight. The samples were then decalcified at 4 °C (pH 7.3) for 10 days in a decalcification solution (1.45% (w/v) EDTA, 1.25% (w/v) NaOH, and 1.5% (v/v) glycerol). After being embedded in paraffin, the bone tissue was sectioned into 5 μm sections with a paraffin microtome (RM2125RTS, Leica Biosystems, Wetzlar, Germany). Deparaffinized tissue sections were washed with PBS and blocked with 3% (w/v) bovine serum albumin (BSA) and 0.4% (v/v) Triton X-100. Then, sections were incubated for 16 h at 4 °C with the following primary antibodies: runt-related transcription factor 2 (Runx2, Product No.: A2851, dilution rate: 1:100, ABclonal Technology Co., Ltd., Wuhan, Hubei, China), osteocalcin (Ocn, Product No.: A6205, dilution rate: 1:150, ABclonal), and β-catenin (Product No.: 8480, dilution rate: 1:150, Cell Signaling Technology Inc., Danvers, MA, USA). The primary antibodies were removed by PBS, and the sections were incubated with secondary antibodies (Product No.: 8114, dilution rate: 1:150, CST) for 1 h at room temperature (RM). The secondary antibody was removed by PBS, and sections were incubated with DAB (3,3′-diaminobenzidine) and hematoxylin for double staining. The sections were dehydrated, covered with coverslips, and photographed using a ZEISS microscope (Axio Scope A1, Oberkochen, Germany). The integrated optical density (IOD)/area was used as a parameter to quantify staining intensity using the image pro plus 6.0 software (Media Cybernetics, Rockville, MD, USA). The images were taken at ×10 magnification.

### Immunofluorescence

DE(1 and 10 μM)were treated with the C3H/10T1/2 cells for 12 h. The C3H/10T1/2 cells were fixed using 4% paraformaldehyde. β-catenin (51067-2-AP, 1:200, proteintech) and labeled with secondary antibodies for 1 h in the dark. After labeling, cells were incubated with DAPI for 5 min. Images were acquired with an Leica confocal microscope (STELLARIS 5, Leica, German). The β-catenin nuclear translocation were mesured.

### Hematoxylin *and* eosin (H&E) staining

The modified hematoxylin and eosin (H&E) staining was performed on deparaffinized bone tissue sections. For H&E staining evaluation, sum area of the trabecular positive region (red area) per maximum image was used in each distal femur as a parameter to quantify the trabecular area using the IPP 6.0 software. The images were taken at ×10 magnification.

### Frozen sections and tissue alkaline phosphatase (ALP) staining

The bone samples were removed from euthanized mice and fixed for 24 h at 4 °C with the solution containing 8% (w/v) paraformaldehyde, L-Lysine, and sodium periodate. Then, tissue specimens were decalcified for 10 days at 4 °C and dehydrated in 30% (w/v) sucrose for 24 h at RM. Finally, samples were prepared as 5 μm sagittal-oriented sections using a Leica frozen microtome (CM1850, Leica, Wetzlar, Germany). The sections were incubated with ALP solution from the ALP staining kit (C3206, Beyotime Institute of Biotechnology, China). For ALP staining, the sum of positive cells was quantified per maximum image in each distal femur using the IPP 6.0 software. The images were taken at ×10 magnification.

### Cell culture and osteoblast differentiation

C3H/10T1/2 cells (a murine embryo fibroblast cell line, classic cell model of mesenchymal stem cells (MSCs)) were purchased from Stem Cell Bank (Code number: SCSP-506, Chinese Academy of Sciences, Shanghai, China). Cells were cultured in MEM and 10% FBS at 37 °C with 5% CO_2_ for subculture. When cells reached 80% confluence, they were subcultured into the osteogenic medium with 10% FBS (MEM supplemented with 0.1 μmol/L dexamethasone, 10 mmol/L β-glycerol phosphate, and 50 μmol/L ascorbic acid). At the same time, DE (1 and 10 μM) was added to the osteogenic medium. The cells were maintained in the osteogenic medium for 1 week, and the medium changed every 3 days. Human Bone Marrow-Mesenchymal Stem Cells (hBMSC) were purchased from Guangzhou Jennio Biotech CO., Ltd. Cells were cultured in DMEM and 10% FBS at 37 °C with 5% CO_2_ for subculture. When cells reached 80% confluence, they were subcultured into the osteogenic medium with 10% FBS (DMEM supplemented with 0.1 μmol/L dexamethasone, 10 mmol/L β-glycerol phosphate, and 50 μmol/L ascorbic acid). At the same time, DE (0.1 and 1 μM) was added to the osteogenic medium. The cells were maintained in the osteogenic medium for 1 week, and the medium changed every 3 days.

### Cell proliferation assay

Cell proliferation was determined using a CCK-8 assay kit (C0037, Beyotime Institute of Biotechnology, China), following the manufacturer’s protocol. A density of 5 × 10^4^ cells/well was used to seed C3H/10T1/2 cells and hBMSC into 96-well plates. After treating the cells with DE (0.01–100 μM) for 7 days, a CCK-8 reagent was added to the wells and incubated at 37 °C for 4 h. Cell proliferation was measured by a microplate reader measuring the optical density absorbance at 450 nm.

### Cellular ALP staining

Cells were fixed in 4% paraformaldehyde for 10 min at RM. After washing with PBS, C3H/10T1/2 cells were incubated with ALP staining buffer following the manufacturer’s protocol. To remove excess dye, cells were washed in PBS. IPP 6.0 software was used to measure ALP-positive cells pictured by microscope.

### Western blotting (WB)

Cells treated with DE in the osteogenic differentiation medium for 7 days were then lysed immediately for 10 min in an SDS-loading buffer (62.5 mM Tris-HCl, 50 mM dithiothreitol, 2% (w/v) sodium dodecyl sulfate, 10% (v/v) glycerol, 0.01% (w/v) bromophenol blue, and pH 6.8) at 96 °C. The primary antibodies, i.e., osterix (Osx, AV31622, 1:500, Sigma), Runx2 (A2851, 1:2000, Abclonal), Osetocalcin(Ocn, ab93876, 1:500, Abcam, Cambridge, UK), GSK-3β (22104-1-AP, 1:500, Proteintech Group Inc., Wuhan, Hubei, China) and P-GSK-3β (Tyr216) (05-413, 1:1000, Merk, Darmstadt, German) were incubated at 4 °C overnight. The secondary anti-rabbit antibody was incubated for 1 h at RM, then visualized using western lighting plus enhanced chemiluminescence (0RT2755, 0RT2655, Perkin Elmer, Waltham, MA, USA). The quantitative gray value of WB bands was measured by ImageJ analysis software (National Institutes of Health, Bethesda, MD, USA).

#### shRNA transfection

For the shRNA transfection assay, C3H/10T1/2 cells were transiently transfected with shRNA (β-catenin) using METAFECTENE® PRO (T040-1.0, biontex, Munich, German) following the shRNA transfection manufacturer’s protocol. The efficiency of transfection was measured by WB. The shRNA (β-catenin) was obtained from GenePharma (GenePharma Co., Ltd., Suzhou, Jiangsu, China), and the sequences were: sense: 5′- CCA​TTG​TTT​GTG​CAG​CTG​CTT-3’.

### Quantitative polymerase chain reaction analysis

*In vivo* experiment, C3H/10T1/2 cells were rapidly extracted using RNAiso Plus and reverse-transcribed into cDNA using the PrimeScript™ RT reagent kit. Quantitative polymerase chain reaction (PCR, RR420A, TaKaRa) analysis was performed with specific gene primers (Table 1) using the Thermal Cycler PCR System (ABI 9700, Applied Biosystems, Foster City, CA, USA). Quantifying RNA expression levels was performed using the 2^−ΔΔCT^ method, and *Gapdh* was used as a positive control.

**TABLE 1:** Primer sequences used in quantitative PCR.

Target gene	GenBank accession no.	Sequences (5′- 3′)	Product size
*GAPDH*	NM_017008.4	Forward, CAG​GGC​TGC​CTT​CTC​TTG​TG; reverse, GAT​GGT​GAT​GGG​TTT​CCC​GT	172 bp
*Osx*	NM_001300837.2	Forward, ACC​TGT​CCT​GTC​CTT​CTG​AG; reverse, ACC​TGT​CCT​GTC​CTT​CTG​AG	119 bp
*Runx2*	NM_001145920.2	Forward, AAT​TAA​CGC​CAG​TCG​GAG​CA; reverse, CAC​TTC​TCG​GTC​TGA​CGA​CG	70 bp
*Ocn*	NM_001037939.2	Forward, TCT​ATG​ACC​TGC​AGA​GGG​CT; reverse, ATA​GCT​CGT​CAC​AAG​CAG​GG	223 bp
*Col1α1*	NM_000088.3	Forward, AGT​GGT​TTG​GAT​GGT​GCC​AA; reverse, GCA​CCA​TCA​TTT​CCA​CGA​GC	170 bp

### Statistical analysis

All data were analyzed using GraphPad Prism version 9.0 software (GraphPad Software Inc., La Jolla, CA, USA). One-way analysis of variance and Tukey multiple comparison test were used to analyze the multiple comparisons in our data. The value was presented as the mean ± SD. The significance level was set at *P* < 0.05.

## Results

### The concentration at which cell proliferation is not affected by DE *in vitro*

CCK-8 assays were performed (Figure 1b) to determine if DE (Figure 1a1-a2) affected the proliferation of C3H/10T1/2 cells and hBMSC. In an ordinary complete medium for 7 days, DE did not significantly affect the growth of C3H/10T1/2 cells at concentrations ranging from 10 nM to 50 μM, or that of hBMSCs from 100 nM to 10 μM.

### DE increased the level of osteogenic proteins *in vitro*

C3H/10T1/2 cells and hBMSCs were cultured for 7 days in osteogenic differentiation medium supplemented with DE at concentrations of 1μM/10 μM and 0.1μM/1 μM, respectively. The ALP staining assay (Figure 1c-f) showed that DE (1 μM and 10 μM)/DE (0.1 μM and 1 μM) increased ALP positivity of C3H/10T1/2 cells/hBMSC in a dose-dependent manner (Figure 1c1-c3, d1-d3/1e1-e3,f1-f3). Based on the quantitative analysis of ALP positive cells (Figure 1g), DE (1 μM and 10 μM) stimulated ALP activity dose-dependently both in C3H/10T1/2 cells and hBMSC. DE (1 μM and 10 μM) was found to promote Runx2, Ocn, and Osx expressions associated with osteogenesis in C3H/10T1/2 cells (Figure 1h). DE (0.1 μM and 1 μM) was found to promote Runx2 and Osx expressions associated with osteogenesis in hBMSC (Figure 1h). The gray value of WB bands was measured as a measure of protein expression level, and results of Runx2, Ocn and Osx (Figure 1i) increased gradually with increasing DE concentrations, peaking at 10 μM with C3H/10T1/2 cells. The gray value of WB bands was measured as a measure of protein expression level, and results of Runx2 and Osx (Figure 1j) increased gradually with increasing DE concentrations, peaking at 10 μM with hBMSC. *In vitro*, DE promotes osteoblast differentiation.

### DE increased the transcription of osteogenic genes *in vitro*

**FIGURE 2:**
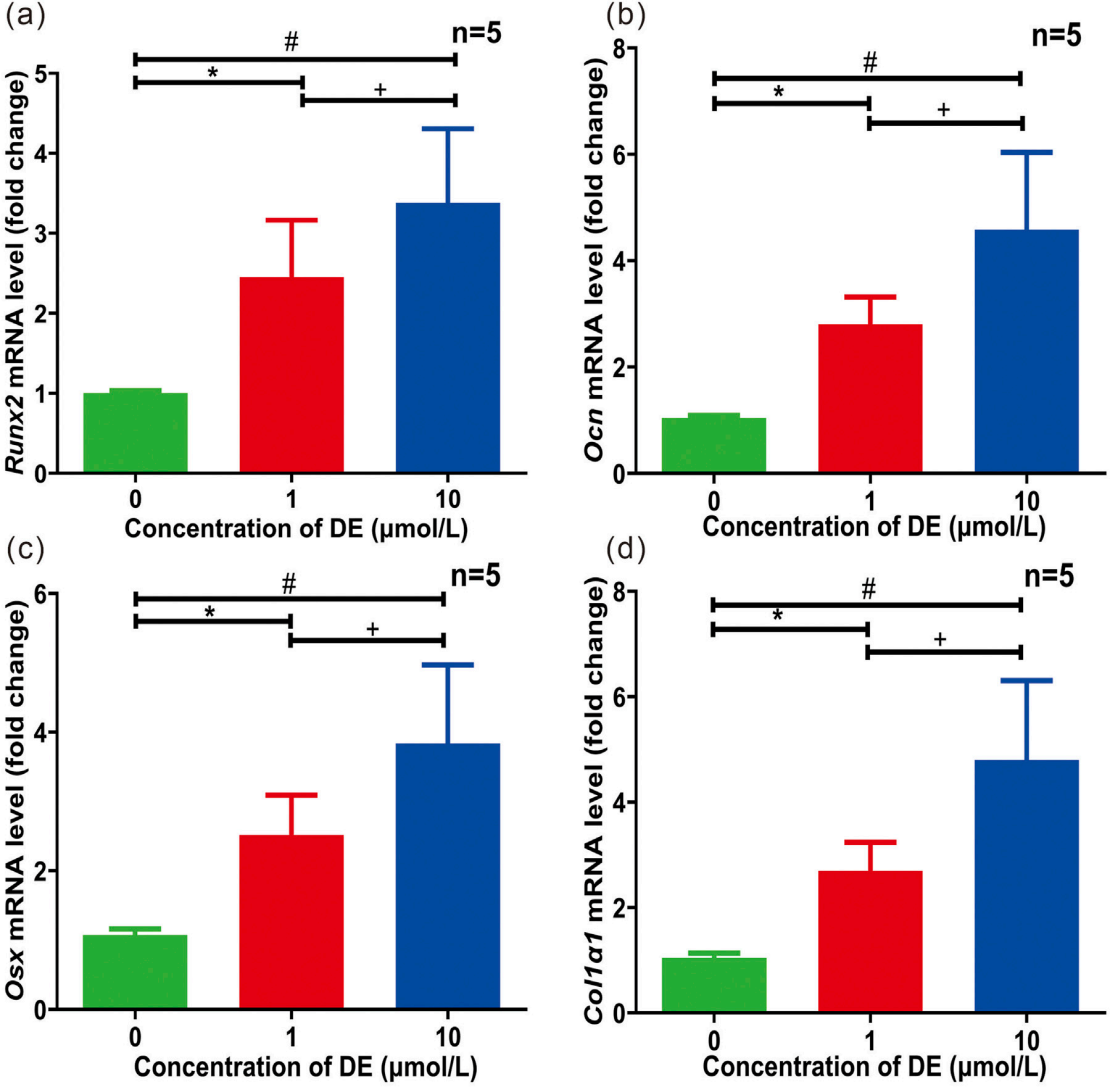
Effects of DE on mRNA transcription of Runx2, Ocn, Osx, and Col1α1 in osteogenesis-induced cultured C3H/10T1/2 cells for 7 days. Quantitative PCR analysis of Runx2 **(a)**, Ocn **(b)**, Osx **(c)**, and Col1α1 **(d)** was performed with the exposure of DE (1 μM and 10 μM) for 7 days of osteogenesis-induced culture. Each sample was measured in triplicate, and the experiment was repeated twice with similar results. *P < 0.05 versus group without DE; +P < 0.05 versus 1 μM DE group; #P < 0.05 versus 10 μM DE group. Values were shown as mean ± SD data from independent experiments **(a–d)**.

We investigated the effects of DE on mRNA levels of osteogenic-related genes (*Runx2*, *Ocn*, *Osx*, and *Col1α1*) in osteogenesis-induced cultured C3H/10T1/2 cells for 7 days. Quantitative PCR analysis of *Runx2* (Figure 2a), *Ocn* (Figure 2b), *Osx* (Figure 2c), and *Col1α*1 (Figure 2d) was performed, and total mRNA levels of osteogenic-related genes were increased in dose-dependent relationship with the exposure of DE (1 μM and 10 μM). As a result, the above results indicate that DE can effectively enhance osteogenic-related gene transcription *in vitro*.

### DE increased the bone mass and osteogenic-related biomarkers *in vivo*

Female C57/BL6 mice were ovariectomized and administered DE intragastrically or E2 via intraperitoneal injection for 1 month. Body weight (Supplementary Figures S1a,c) and uterus mass (Supplementary Figures S1b,d) were recorded in order to confirm the effects of ovariectomy validated by increased. In the mice, OVX was successfully body weight and decreased in uterus weight. An effect depending on the dose of DE or E2 on bone formation was evaluated (Supplementary Figure S2a) using the H&E staining assay. In a manner depending on DE dose, the increasing trabecular area was found in the OVX + DE group (10 mg/kg/day and 20 mg/kg/day) compared to the OVX group, with a peak of 20 mg/kg/day. As a positive group, the trabecular area in OVX + E2 was significantly increased compared with OVX group (Supplementary Figure S2a). Our intervention concentration of DE *in vivo* was set at 20 mg/kg/day.

**FIGURE 3:**
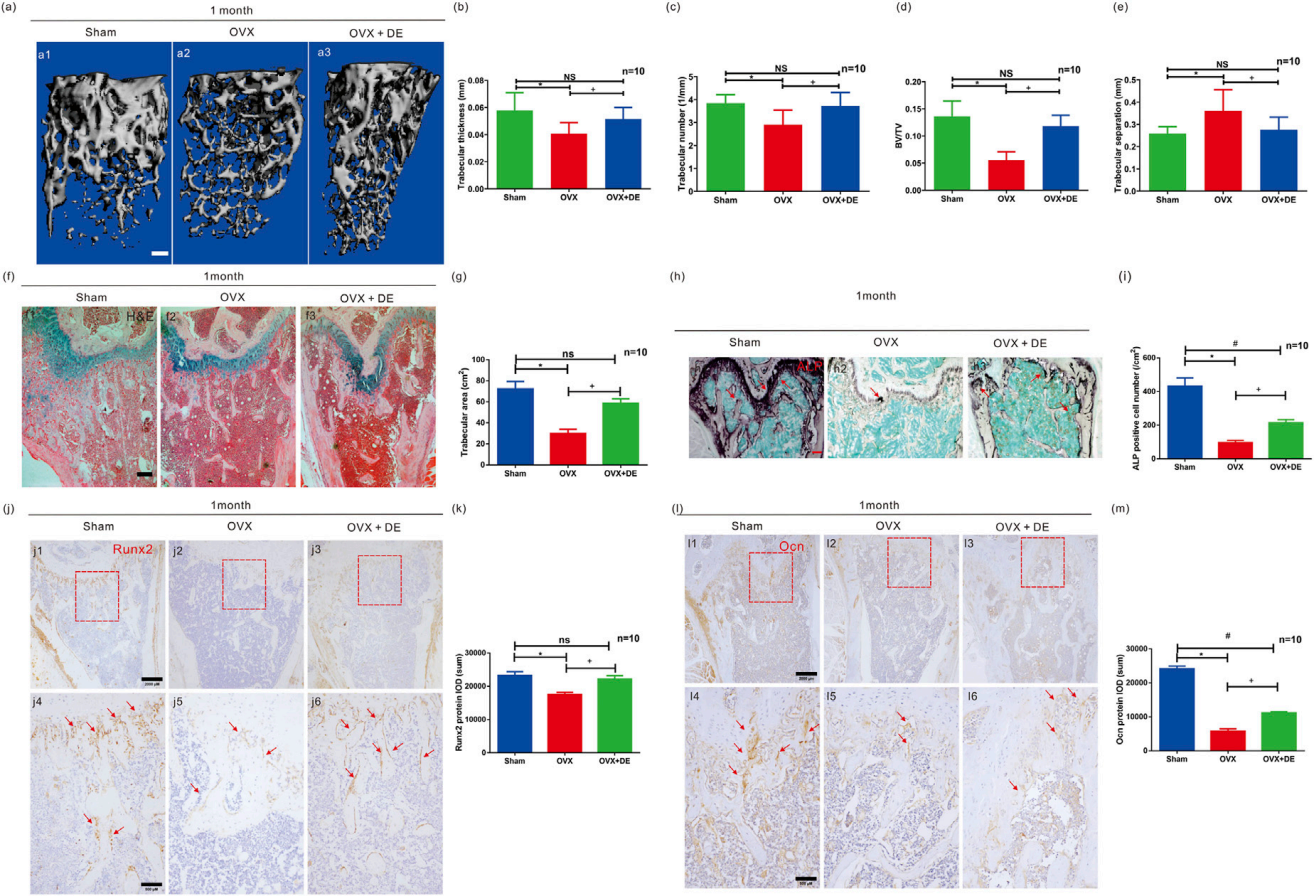
Effects of DE on bone formation biomarkers *in vivo*. The bone metabolism indicators were measured in ovariectomy-induced osteoporosis mice with intragastric administrations of DE (20 mg/kg/day) for 1 month **(a)** Micro-CT scan of the distal femur (a1-a3) was performed, and bone structure indicators were obtained, including trabecular thickness **(b)**, trabecular number **(c)**, BV/TV **(d)** and trabecular separation **(e)** in sham, OVX, and OVX + DE mice. **(f)** The H&E staining of the distal femur (f1-f3) was performed, and the trabecular area was measured in sham, OVX, and OVX + DE group **(g)**. **(h)** The ALP staining of the distal femur was performed in sham, OVX, and OVX + DE mice (h1-h3), and the ALP-positive cells were measured **(i)**. The Runx2 **(j)**, and j1-j6) IHC staining of the distal femur was performed and the sum integrated optical density (IOD) of Runx2 **(k)** was quantified in sham, OVX, and OVX + DE mice. The Ocn **(l)** and l1-l6) IHC staining of the distal femur and the sum IOD of Ocn **(m)** were quantified in sham, OVX, and OVX + DE mice. Quantitative counting of the trabecular area, positive cell and sum of IOD was measured by IPP analysis software. Each group contained ten mice. *P < 0.05 versus sham group; +P < 0.05 versus OVX group; #P < 0.05 versus OVX + DE group; NS:non significance. Values were shown as mean ± SD data from independent experiments **(b-e,g,i,k,m)**.

As part of our investigation of DE’s effect on trabecular bone formation, micro-CT analysis was performed on the mice distal femur in sham, OVX, and OVX + DE (Figure 3a) group. As compared to OVX, OVX + DE mice had significantly higher Tb. Th (Figure 3b), Tb. N (Figure 3d), BV/TV (Figure 3e), and lower Tb. Sp (Figure 3c). H&E staining of the distal femur (Figure 3f) showed an increase in the trabecular area compared to the OVX group (Figure 3g). The distal femur was stained with ALP (Figure 3h). ALP-positive cells were more significant in OVX + DE mice than in OVX mice (Figure 3i). In the distal femur, IHC staining of Runx2 (Figure 3j) was performed, and the results indicated that the sum IOD for Runx2 (Figure 3k) increased in OVX + DE group compared to OVX mice. We conducted IHC staining of Ocn (Figure 3l) on the mice’s distal femur, and the results showed that Ocn’s sum IOD (Figure 3m) increased in OVX + DE group when compared with OVX mice. As a result, DE can prevent osteoporosis in mice induced by ovariectomy.

### DE enhanced osteogenic differentiation via the Wnt/β‐catenin signaling pathway

**FIGURE 4:**
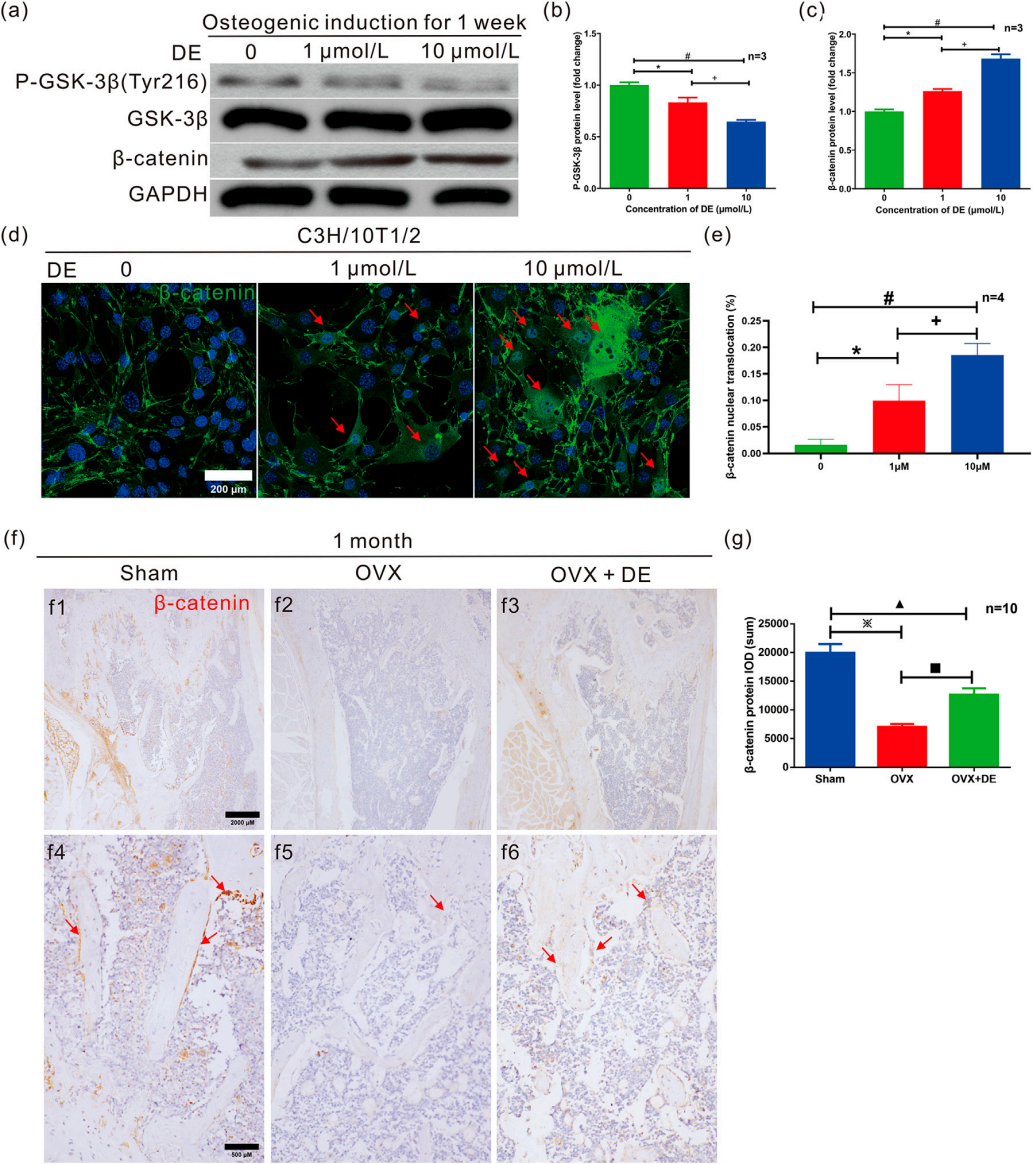
Effects of DE on Wnt/β‐catenin signaling pathway *in vitro* and *in vivo*. The activity level of the Wnt/β‐catenin signaling pathway was measured in the presence of DE in C3H/10T1/2 cells and OVX models. **(a)** The protein level of P-GSK3β (Tyr216) and β-catenin by WB analysis and quantitative protein level of P-GSK3β **(b)** and β-catenin **(c)** were obtained in osteogenesis-induced cultured C3H/10T1/2 cells for 7 days **(d)** The β-catenin IF staining of the C3H/10T1/2 cells was performed, and the β-catenin nuclear translocation (%) was mesured **(e)**. The β-catenin IHC staining of the distal femur **(f)** was performed, and the sum IOD of β-catenin **(g)** was measured in sham, OVX, and OVX + DE mice. Quantitative counting of the sum of IOD was measured by IPP analysis software. The quantitative gray value of WB bands was measured by ImageJ analysis software. Each sample was measured in triplicate, and the experiment was repeated twice with similar results. Each group contained ten mice. *P < 0.05 versus group without DE; +P < 0.05 versus 1 μM DE group; #P < 0.05 versus 10 μM DE group. ※P < 0.05 versus sham group; ■P < 0.05 versus OVX group; ▲P < 0.05 versus OVX + DE group. Values were shown as mean ± SD data from independent experiments **(b,c,e,d,g)**.

**FIGURE 5:**
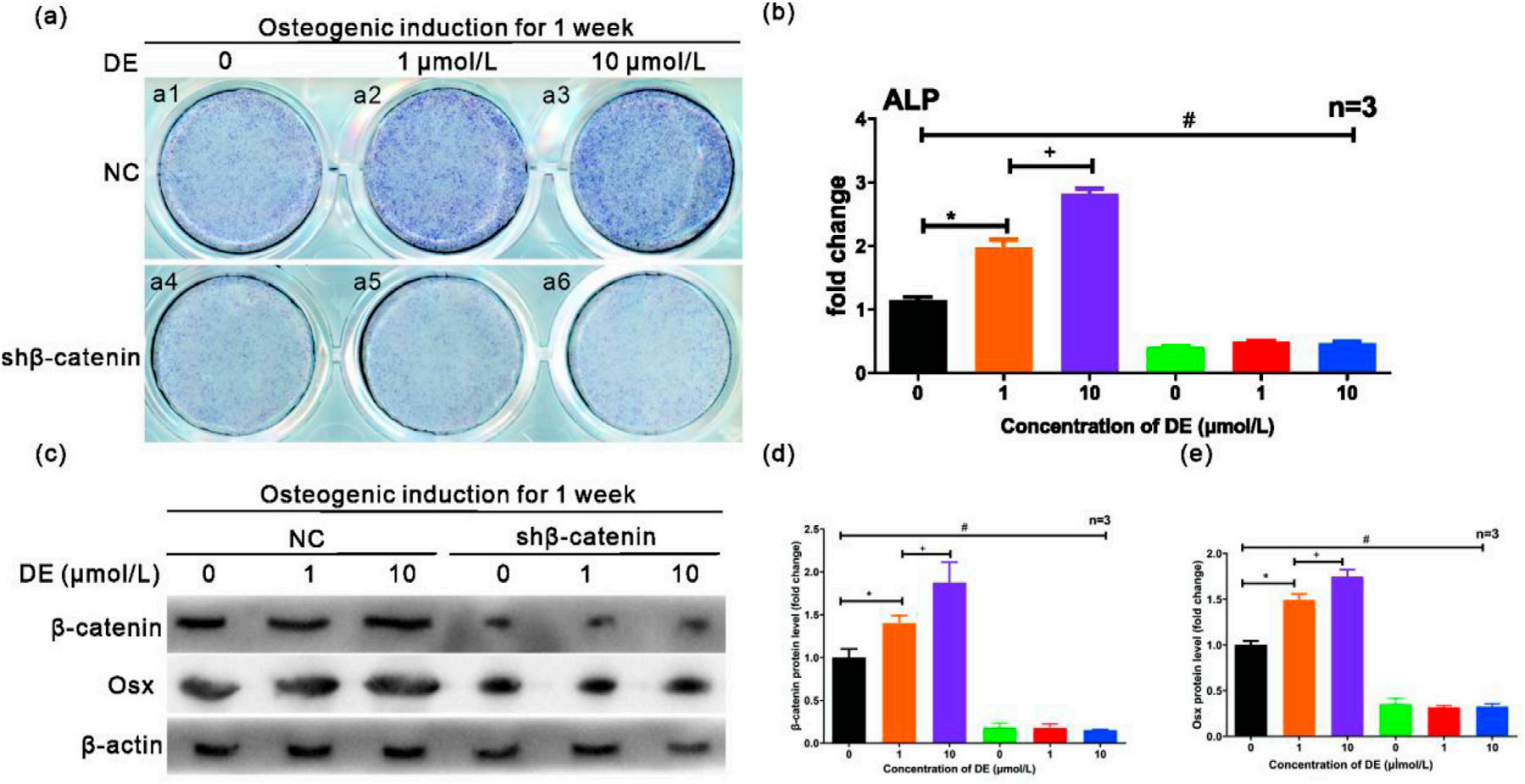
Effects of DE on C3H/10T1/2 cells’ osteogenic differentiation by β‐catenin depletion. C3H/10T1/2 cells were sequentially treated with DE (1 μM and 10 μM) and shRNA of β‐catenin for 7 days with osteogenesis-induced culture. **(a)** The ALP staining was performed, and positive cell counting **(b)** was measured. **(c)** WB measured the β‐catenin and OSX protein levels. The quantitative expression of β‐catenin **(d)** and Osx **(e)** was obtained by administration of DE (1 μM and 10 μM) in C3H/10T1/2 cells. Quantitative counting of the positive cell was measured by IPP analysis software. The quantitative gray value of WB bands was measured by ImageJ analysis software. Each sample was measured in triplicate, and the experiment was repeated twice with similar results. *P < 0.05 versus group without DE; +P < 0.05 versus 10 μM DE group; #P < 0.05 versus 10 μM DE with the shRNA-β‐catenin group. Values were shown as mean ± SD data from independent experiments **(b,d,e)**.

C3H/10T1/2 Cells were treated with DE for 7 days in an osteogenic medium. By WB (Figure 4a), we measured the levels of phosphorylation of GSK-3β (Tyr216) (a hallmark of GSK-3β activity) and β‐catenin. It was found that P-GSK-3β (Tyr216) was decreased and β-catenin levels were increased in osteogenesis-induced cultured C3H/10T1/2 cells with DE (1 μM and 10 μM). Immunofluorescence analysis demonstrated that DE (1 and 10 μM) promoted β-catenin nuclear translocation in C3H/10T1/2 cells (Figure 4d). This effect was confirmed by quantitative analysis (Figure 4e). Furthermore, IHC staining of β-catenin was performed on the mice’s distal femur (Figure 4f). The results indicated that β-catenin sum IODs increased in OVX + DE mice compared to OVX mice (Figure 4g). The critical role of β-catenin was confirmed by its knockdown (Figures 5c,d), which reversed the DE-mediated increases in ALP activity (Figures 5a,b) and Osx protein levels (Figures 5c,e), suggesting that the effect of DE on osteogenic differentiation depends on β-catenin. Based on these data, DE is shown to promote osteogenic differentiation and trabecular bone formation by preventing GSK-3β activity and β-catenin degradation.

## Discussion

During early postmenopausal women with sex steroid deficiency (Compston et al., 2019; Cheung et al., 2016), an imbalance between bone resorption and bone formation causes an accelerated decline in bone mineral density due to a longer lifespan of osteoclasts and a shorter lifespan of osteoblasts (Manolagas, 2010). Due to this, postmenopausal osteoporosis can be alleviated with agents that increase osteoblastogenesis or decrease osteoclastogenesis. Osteoblasts are derived from Bone Marrow Mesenchymal Stem Cells (BMSC) (Kokabu et al., 2016). C3H/10T1/2 cells and hBMSC were differentiate into osteoblasts when treated with appropriate osteogenic induction to analyze the osteogenic effect of DE (Tang et al., 2004; Zhang et al., 2021).

Currently, there are several anti-osteoporosis drugs on the market, but they have potential side effects, poor efficacy, and high prices, so finding a more effective and safer alternative is critical. Numerous *in vivo* and *in vitro* studies have evaluated plant sources as non-pharmaceutical alternatives for bone benefits (Banu et al., 2012). As a result of increasing lumbar spine Bone Mineral Density (BMD) and decreasing urine bone resorption markers, soy isoflavones may prevent osteoporosis and improve bone strength for menopausal women. According to a model of senile osteoporosis, dietary phlorizin increases β-catenin activity by inhibiting GSK-3β and promotes osteoblastogenic bone formation (Antika et al., 2017).

Active ingredients in Chinese medicine are isolated from single herbs or traditional herbal formulas, and their active ingredients are more effective, safer, and easier to use than traditional decoctions. With the development of modern purification technology, Chinese medicine’s active ingredients have become increasingly popular in China, as well as attracting worldwide attention (Lin et al., 2017). *In vitro*, DE, a main bioactive alkaloid of *Dendrobium nobile* Lindl., has been shown to eliminate ROS production, inhibits p38-c-Fos-NFATc1 pathway, and reduces the expression of MMP9. The study demonstrates that DE also attenuates inflammatory bone loss *in vivo* (Deng et al., 2022). In our research, we found that DE could inhibit the trabecular bone loss of OVX mice, via promoting a murine embryo fibroblast cell line osteogenic differentiation and consequent trabecular bone formation, at least partially through the canonical Wnt/β-catenin pathway. To our knowledge, this is the first study to identify DE as a critical promoter of bone formation.

Osteoblastogenesis is regulated by a hierarchical transcriptional cascade (Amarasekara et al., 2018). The master transcription factor Runx2 initiates the differentiation of mesenchymal stem cells (MSCs) into preosteoblasts, which is then followed by the expression of its key downstream target, Osterix (Osx) (Amarasekara et al., 2018). Osx, in turn, drives the expression of markers characteristic of successive differentiation stages: Alkaline Phosphatase (ALP) to mature the extracellular matrix (ECM) and Osteocalcin (Ocn) for final matrix mineralization (Amarasekara et al., 2018). In line with this established pathway, we found that DE treatment dose-dependently upregulated the expression of Runx2, Osx, ALP, and Ocn in C3H/10T1/2 cells or hBMSC. The pro-osteogenic effect of Dendrobine (DE) is conserved across species, from murine cells to human primary cells. Crucially, this pro-osteogenic effect was confirmed *in vivo*, where DE administration restored the levels of ALP, Runx2, and Ocn in OVX mice. Collectively, these results demonstrate that DE promotes osteoblast differentiation in both cellular and animal models.

We therefore conclude that DE promotes osteoblast differentiation *in vitro* and *in vivo* by activating the Wnt/β-catenin axis. Mechanistically, β-catenin acts as a transcriptional co-activator for Runx2, a key early osteogenic regulator. The activation of this axis by DE likely transactivates Runx2, which in turn induces Osterix (Osx) to upregulate downstream markers including ALP and Ocn, thereby orchestrating the entire osteoblastogenic program.

Osteoblastogenesis is regulated by multiple signaling pathways, such as Wnt, transforming growth factor β (TGF-β), parathyroid hormone (PTH), fibroblast growth factor (FGF), bone morphogenetic protein (BMP), and hedgehog (Hh). The canonical Wnt pathway is particularly critical; upon activation, β-catenin translocates into the nucleus to drive the expression of osteogenic genes (Huang et al., 2007). In a previous study, it was found that Dermo1-Cre strategy conditionally knocked out β-catenin in early MSCs condensations and reduced osteoblast differentiation (Day et al., 2005). Our study showed that DE enhanced β-catenin expression and promoted its nuclear translocation in C3H/10T1/2 cells; increased β-catenin expression was also confirmed in osteoblastic cells from OVX mice. Furthermore, knocking down β-catenin abolished the pro-osteogenic effect of DE, demonstrating that its action is dependent on this pathway. This study establishes that the activation of the Wnt/β-catenin axis is the mechanism by which DE enhances osteoblast differentiation in C3H/10T1/2 cells and ameliorates bone loss in OVX mice.

β-catenin regulates *Runx2* transcription by acting as a transcriptional co-activator (Karner and Long, 2017). Wnt/β-catenin signaling activates the Runx2 promoter and promotes endogenous Runx2 expression in pluripotent mesenchymal and osteoprogenitor cells (Gaur et al., 2005). Our findings lead us to propose a coherent mechanism: the upregulation of the Wnt/β-catenin axis by DE transactivates Runx2, which in turn initiates Osx transcription. This sequence ultimately drives the expression of terminal differentiation markers (Ocn and ALP) and enhances osteoblastogenesis.

GSK-3β is a crucial negative regulator of canonical Wnt/β-catenin signaling, suggesting GSK-3β as a potential molecular target for the treatment of osteoporosis (Antika et al., 2017). Upon tyrosine phosphorylation at Tyr216, GSK-3β activity is significantly increased (Varisli et al., 2015). Our study found that DE dose-dependentl suppressed the phosphorylation of GSK-3β at Tyr216 in C3H/10T1/2 cells, suggesting that DE may increase Wnt/β-catenin pathways by inhibiting GSK-3β.

## Conclusion

In summary, our results demonstrate that DE promotes osteoblastogenesis in MSCs and enhances bone formation to attenuate the bone loss in OVX mice. This effect is mediated through the activation of the canonical Wnt/β-catenin pathway via inhibition of GSK-3β activity. According to the study, DE can be a candidate treatment to prevent bone loss in postmenopausal osteoporosis.

## Data Availability

The original contributions presented in the study are included in the article/Supplementary Material, further inquiries can be directed to the corresponding authors.

## Abbreviations

hBMSC, human bone marrow-derived mesenchymal stromal cells; E2, estrogen; micro-CT, Micro-computed tomography; IHC, Immunohistochemical; BSA, bovine serum albumin; H&E, hematoxylin and eosin; BSA, bovine serum albumin; WB, Western blotting; RM, room temperature; DE, Dendrobine; GDM, Diabetes mellitus; OVX, Ovariectomy; Tb. Th, Trabecular thickness; BV/TV, Bone volume/tissue volume; Tb. N, Trabecular number; Tb. Sp, Trabecular separation; IOD, Integrated optical density; ALP, Alkaline phosphatase; CCK-8, Cell Counting Kit-8; MSCs, Mesenchymal stem cells; Runx2, Runt-related transcription factor 2; Ocn, Osteocalcin; Osx, Osterix; ECM, Extracellular matrix; Hh, Hedgehog; TGF-β, Transforming growth factor β; PTH, Parathyroid hormone; FGF, Fibroblast growth factor; BMP, Bone morphogenetic protein.
