# Supplementary material for: Dendrobine promotes bone formation via the canonical Wnt/β-catenin signaling pathway and prevents postmenopausal osteoporosis
Source: Front Pharmacol. 2025 Dec 3;16:1616070. doi: 10.3389/fphar.2025.1616070 (PMC12709085; doi:10.3389/fphar.2025.1616070)
Supplement: Supplementary file 1 [file DataSheet1.pdf]

Supplemental Figures

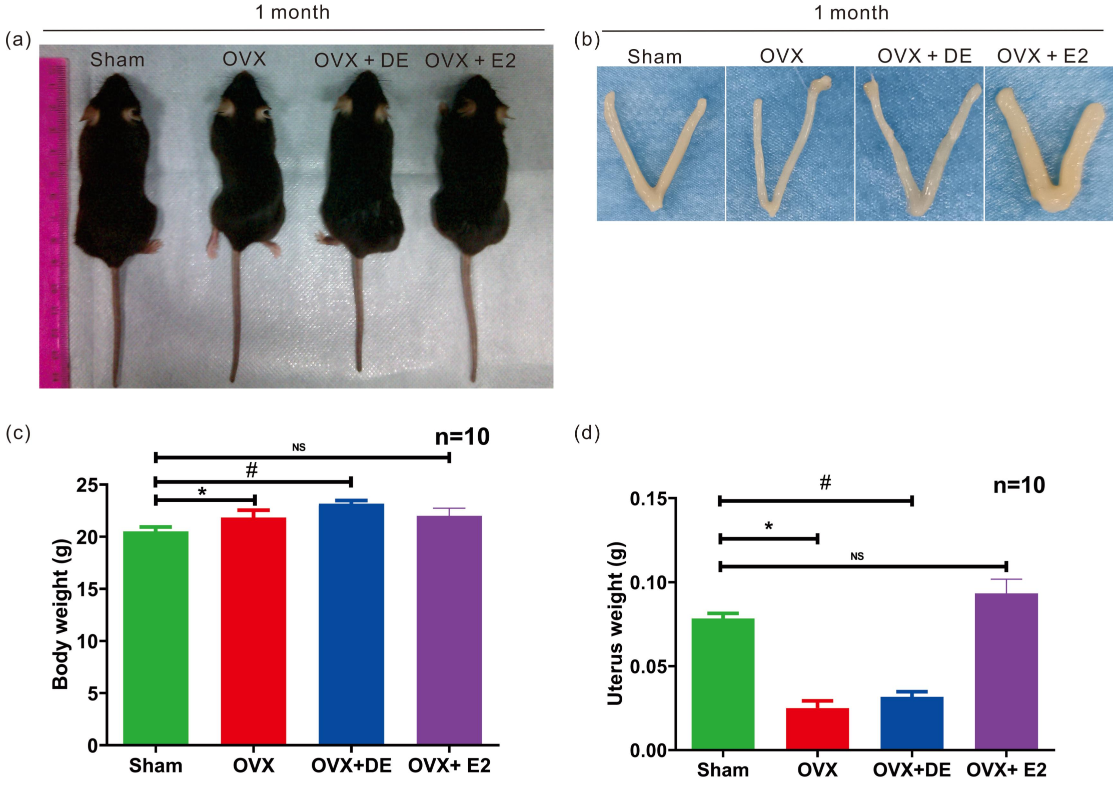

Supplementary Figure 1. Ovariectomize was successfully performed in the mice.

(a) The body and uterus appearance (b) was obtained, as well as body (c) and uterus (d) weight were measured with 1 month of intragastric administrations of DE (20 mg/kg/day) or intraperitoneally injection of E2 (5mg/kg/day). Each group contained ten mice. *P<0.05 versus OVX group; #P<0.05 versus OVX+ DE group. Values were shown as mean ± SEM data from independent experiments (c and d).

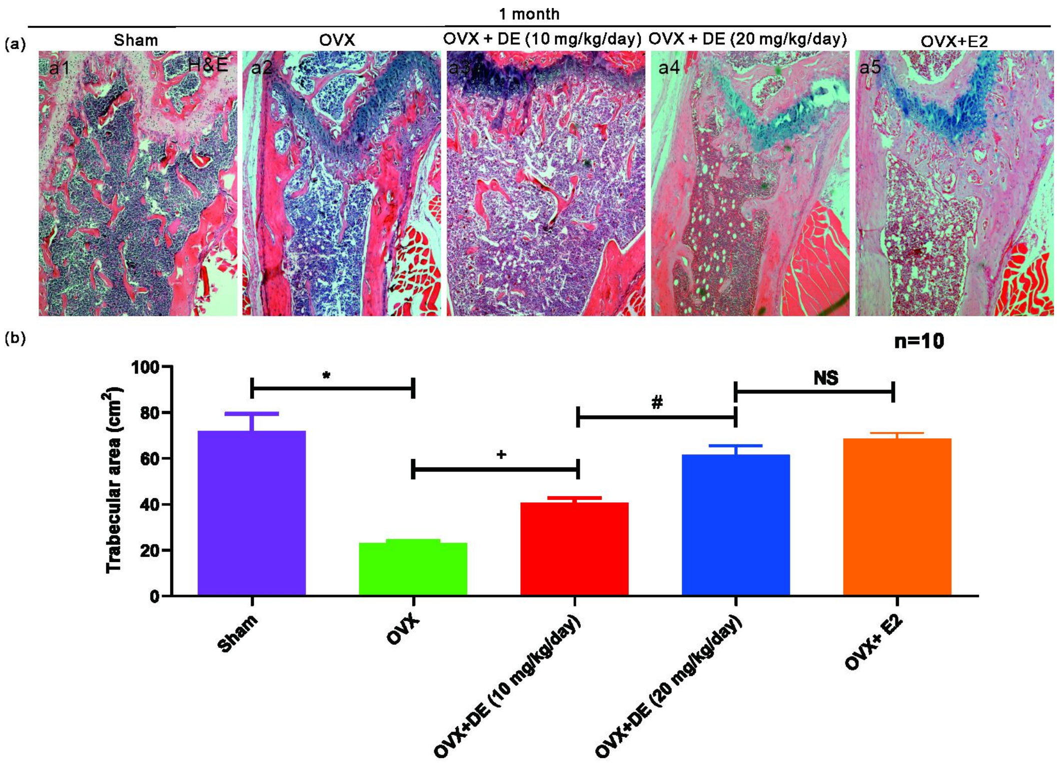

Supplementary Figure 2. Effect of different concentrations of DE and E2 on bone formation in different group mice.

(a) The H&E and trabecular area (b) were performed in the distal femur in the sham, OVX, OVX + DE, and OVX +E2 groups (used as a positive control). Quantitative counting of the trabecular area and positive cell were measured by IPP analysis software. Each group contained ten mice. *P<0.05 versus sham group; +P<0.05 versus OVX group; #P<0.05 versus OVX+ DE (10 mg/kg/day) group; NS: non significance. Values were shown as mean ± SEM data from independent experiments.
